# Microglia as Therapeutic Target for Radiation-Induced Brain Injury

**DOI:** 10.3390/ijms23158286

**Published:** 2022-07-27

**Authors:** Qun Liu, Yan Huang, Mengyun Duan, Qun Yang, Boxu Ren, Fengru Tang

**Affiliations:** 1 The School of Basic Medicine, Health Science Center, Yangtze University, Jingzhou 434023, China; liuqunq1998@163.com (Q.L.); huangyan9808@126.com (Y.H.); 2 Department of Pharmacology, School of Medicine, Yangtze University, Jingzhou 434023, China; mengyun_duan@163.com (M.D.); yangqun516021@yangtzeu.edu.cn (Q.Y.); 3 Radiation Physiology Laboratory, Singapore Nuclear Research and Safety Initiative, National University of Singapore, Singapore 138602, Singapore

**Keywords:** microglia, brain injury, ionizing radiation, cognitive effects, therapy

## Abstract

Radiation-induced brain injury (RIBI) after radiotherapy has become an increasingly important factor affecting the prognosis of patients with head and neck tumor. With the delivery of high doses of radiation to brain tissue, microglia rapidly transit to a pro-inflammatory phenotype, upregulate phagocytic machinery, and reduce the release of neurotrophic factors. Persistently activated microglia mediate the progression of chronic neuroinflammation, which may inhibit brain neurogenesis leading to the occurrence of neurocognitive disorders at the advanced stage of RIBI. Fully understanding the microglial pathophysiology and cellular and molecular mechanisms after irradiation may facilitate the development of novel therapy by targeting microglia to prevent RIBI and subsequent neurological and neuropsychiatric disorders.

## 1. Introduction

Radiotherapy is the mainstay of first-line treatment in primary and metastatic brain tumors [1]. Unfortunately, irradiated areas always include normal brain tissue that surrounds the tumor, and as a result, many patients experience progressive and irreversible side effects. At the early stage after radiotherapy, patients may have transient, self-healing symptoms, including headache, lethargy, fatigue, and attention deficits, whereas more than 50% of oncology patients who survive more than 6 months after whole-brain radiation develop irreversible cognitive impairment [2,3,4,5]. The molecular and cellular mechanisms behind these effects are complex, involving the production of proinflammatory factors from microglia, cascades of signal transduction, gliosis, altered neurogenesis, and injury of endothelial cells (ECs) [6,7]. Currently, multiple radiation strategies that limit normal tissue toxicity such as hippocampal avoidance radiotherapy, proton beam therapy, and ultra-high-dose-rate irradiation, have been shown to moderate RIBI in clinical or preclinical studies [8,9,10,11]. However, the lack of understanding of cellular responses to ionizing radiation (IR) in the central nervous system (CNS) has limited the development of new therapeutic approaches.

**Figure 1:**
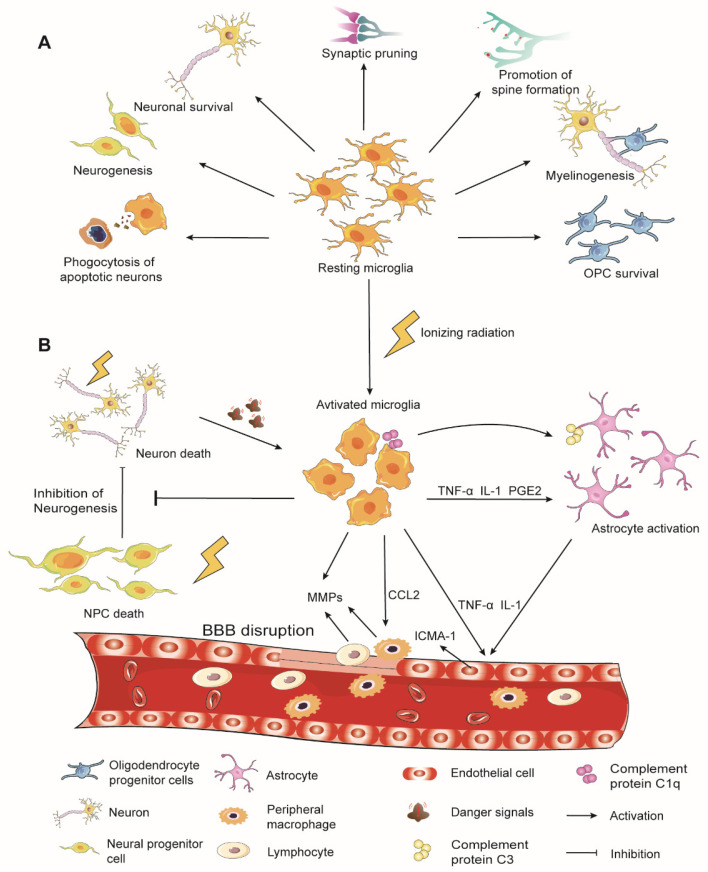
Overview of the role of microglia in healthy brain and radiation-induced brain injury (RIBI). (**A**) Physiological functions of microglia in healthy brain. (**B**) The interaction of activated microglia with central nervous system (CNS) cell populations mediates the development of RIBI. Ionizing radiation (IR) directly induces microglial activation, while irradiated neurons and endothelial cells (ECs) release “danger” signals (high mobility group box 1, adenosine triphosphate, uridine diphosphate, and so on) to exacerbate microglial activation. By participating in complement cascade and secreting pro-inflammatory mediators such as TNF-α, interleukin-1 (IL-1), and prostaglandin E2 (PGE2), activated microglia can trigger the astrocytes activation, prevent neurogenesis and neural progenitor cell (NPC) differentiation, and stimulate the expression of intercellular adhesion molecule 1 (ICAM-1) in endothelial cells. ICAM-1 accumulation and blood–brain barrier (BBB) damage induced by IR cause the increased infiltration of peripheral immune cells, and this is exacerbated by monocyte chemoattractant protein-1 (MCP-1/CCL2) secreted by microglia. Subsequently, matrix metalloproteinases (MMPs) secreted by microglia and infiltrating cells further aggravate BBB damage.

Microglia exert various effects in brain development and homeostasis, including neurogenesis, phagocytosis of dying or apoptotic neurons, synaptic pruning, formation of neural circuits, and myelinogenesis (Figure 1A) [12]. Under physiological conditions, microglia are in a resting state, as neurons express a range of inhibitory factors, such as CD200, CX3CL1, CD22, CD47, and CD55 [13,14,15]. They often display a ramified morphology characterized by small somas and elongated processes and monitor the surrounding microenvironment with constantly moving processes in all directions [16]. Upon stimulation of the CNS by insult such as IR exposure, microglia are in an activated state. Although the phagocytosis of dead cells by activated microglia is vitally important for maintaining the brain microenvironment homeostasis, persistent activation leads to chronic neuroinflammation and cognitive impairment in late RIBI [17,18].

Traditionally, dichotomous classification was applied to microglia, and activated microglia was categorized as either in the M1 or M2 state, which represent pro- and anti-inflammatory states, respectively [19]. Microglia are considered to be in the M1 state after high-dose radiation exposure. They assume an amoebic morphology with larger soma and shorter protrusions, accompanied by enhanced phagocytosis and the release of a variety of proinflammatory mediators such as interleukin 1β (IL-1β), IL-6, reactive oxygen species (ROS), tumor necrosis factor-α (TNF-α), etc [17,20]. M2 microglia phagocytose dead cells and produce anti-inflammatory cytokines and neurotrophic factors. The M1/M2 definitions were derived from cultured microglia stimulated by a single cytokine in vitro, which fails to reflect the subtler phenotype of microglia in vivo. Hence, based on the microglial functional endpoints, rather than on transient morphological changes or microglial activation markers, we used terms such as “neuroprotective” and “neurodestructive” to describe the different functional activation states of microglia in this review paper. Notably, better ways to define microglial activated states should consider not only different exogenous or endogenous stimuli such as trauma, infection, tumors, neurodegeneration, etc., but also differences inherent to the organism, including but not limited to age, sexual dimorphism, regional heterogeneity, and functional status of the nervous system [19]. In this review, we described the classical and more recent studies of the contribution of microglia to RIBI, in particular, the molecular mechanisms by which microglia mediate secondary injury after RIBI. We then highlighted the potential therapeutic targets that regulated microglia activation and functional diversity, and based on these findings, we discussed major obstacles for preclinical studies and clinical translation.

## 2. Overview of Microglia Physiological Functions

Fate mapping revealed that microglia originated from myelo-erythroid progenitor cells in the mammalian yolk sac. These progenitor cells migrate into the brain between embryonic day 9.5 and 14.5 in mice [21,22,23]. In humans, microglia begin to invade and colonize in the brain around 4.5 to 5.5 weeks of gestation, prior to the formation of the vascular system and blood–brain barrier (BBB), and the development of astrocytes and oligodendrocytes [24]. In contrast to macrophages in other tissues, microglia density remains stable over the lifetime of mice and humans, which relies on microglia local proliferation. They renew themselves at a low rate via coupled apoptosis and proliferation without the supplement of circulating progenitors from blood or bone marrow [25,26]. In addition, microglia generation is dependent on multiple cytokines such as colony stimulating factor 1 (CSF-1), IL-34, and IL-1 [27,28], as well as transcription factors PU.1 and interferon regulatory factor 8 (IRF8) [29].

The heterogeneity of microglia in terms of temporal, spatiality, and gender has been extensively studied. Nowadays, the application of bulk RNA-sequencing (RNAseq) and single-cell RNAseq in transcriptomics further indicates diversity in microglia gene characteristics due to regional microenvironment and age differences [30,31]. Studies at the microglial transcriptome level have revealed distinct gene expression profiles and functional characteristics at different stages of brain development [32,33]. For example, early microglia exhibit an amebic morphology akin to the activated states and are enriched for a variety of expressed genes associated with cell proliferation, DNA replication, and lysosome, which may reflect the enhancement of microglia proliferation and synaptic pruning at the embryonic and early postnatal stage [31,33,34]. In contrast, single-cell RNAseq exhibits less than 20 genes differentially expressed by adult microglia distributed across different brain regions, implying the homogeneity of adult steady-state microglia [32].

During embryogenesis, microglia participate in shaping functional structures of neuronal networks by removing excess neurons and guiding neuronal migration [12]. Adult microglia maintain the homeostasis of the adult neurogenic niches by phagocytosis of apoptotic neurons in the subgranular zone (SGZ) of the dentate gyrus (DG) that are not integrated into the hippocampal circuit [35]. Partial ablation of microglia reduced the survival ratio of neuroblasts in the DG [36]. Neurotrophic factors derived from microglia, such as brain-derived neurotrophic factor (BDNF) [37], transforming growth factor-β (TGF-β) [38], and insulin-like growth factor-1 (IGF-1) [39,40], play an irreplaceable role in neurogenesis and neuron survival. In particular, IGF-1 promoted neurogenesis in the SVZ and maintained the survival of cortical neurons in cerebral ischemic injury, while the administration of IGF-1 inhibitors caused significant death of cortical neurons [40,41]. CX3CR1 is a chemokine receptor belonging to the G protein-coupled receptor family and is almost exclusively expressed by microglia in the CNS [42]. The disruption of fractalkine (FKN)/CX3CR1, as one of the signaling pathways for microglia–neuron communication, reduced the survival and proliferation of neural progenitor cells. This aftermath possibly results from the fact that CX3CR1 blockade or knockout alters the generation of neurogenesis-promoting cytokines from microglia [43,44]. In addition, a study of SVZ microglia suggested that depletion of microglia impairs migration of neuroblasts to the olfactory bulb through a rostral migratory stream [45].

Microglia with robust phagocytosis shape neural networks by eliminating redundant synapses selectively through axonal and dendritic pruning during postnatal brain development [46,47]. Synaptic components have been found in the cytoplasm of microglia, especially in lysosomes [47,48]. More importantly, with the observation of slices of organotypic hippocampal cultures with focused ion beam scanning electron microscopy, time-lapse images show processes by which microglia actively engulf synaptic components [49]. The complement system is typically involved in synaptic pruning. Complement protein C1q initiates complement cascade and C3 aggregates at redundant synapses, followed by phagocytosis of labeled synapses by microglia expressing complement receptor-3 (CR3) [48]. Wang et al. reported that in the hippocampus of adult mice, microglial involvement in the elimination of remote memory-related synapses is mediated by the complement pathway [50]. Depletion of microglia by diphtheria toxin or PLX3397 or inhibition of the classical and alternative complement pathways by CD55 significantly improved memory amnesia in mice subjected to contextual fear conditioning, suggesting that elimination of synapses via the microglial complement pathway exacerbates memory amnesia [50]. In addition to the complement system, microglial surface receptors CX3CR1 and triggering receptor expressed on myeloid cells 2 (TREM2) have a positive effect on synaptic pruning, which is inhibited in mice with relevant gene knockout [47,51,52].

In contrast to synaptic pruning, microglia also promote synapse formations. In the mouse somatosensory cortex of the first two postnatal weeks, the contact of microglia with dendritic spines induced Ca^2+^ transients and actin accumulation at the contact sites, which promoted the formation of filopodia that were required for synaptogenesis [53]. In adult mice, neuron-derived IL-33 can incite microglia to remodel the extracellular matrix, thereby enhancing synaptic formation and memory consolidation [54]. Microglia also release IL-10 to increase the number of dendritic spines of hippocampal neurons, as well as BDNF to promote learning-dependent synapse formation [55,56]. Genetic depletion of BDNF from microglia of mice lessened synapse remodeling and caused deficits in multiple learning tasks [56]. In short, the effects of microglia on synapses are bidirectional. They not only promote formations of functioning synapses but also remove superfluous synapses, shape neural circuits, and therefore affect advanced brain function such as memory and learning.

For myelinogenesis, microglia create a microenvironment conducive to oligodendrocyte progenitor cell (OPC) recruitment and maturation by phagocytosing myelin debris, modifying the extracellular matrix, and secreting IGF1, IL-1β, activin A, and galectin 3 [57,58,59,60]. Before and after myelination, clearance of dead and partially surviving oligodendrocytes in a microglial phagocytosis-dependent manner maintains proper myelination [59,61]. In a neuronal activity study in zebrafish, microglial, under the regulation of neuronal activation, also specifically engulf myelin sheaths [57]. Microglial depletion or phagocytosis inhibition by knockdown CX3CR both increase the incidence of ectopic myelin, abnormal myelin structure, and excessive OPC population [57,61].

## 3. Microglia in Radiation-Induced Brain Injury

Following IR, microglia sense microenvironmental changes immediately and react accordingly. By interacting with neurons, ECs, astrocytes, and oligodendrocytes, these cells mediate brain pathogenesis, including BBB disruption, infiltration of peripheral immune cells, neuronal death, inhibition of neurogenesis, and structural damage of synapses (Figure 1B) [62,63,64,65].

### 3.1. Microglial Activation

DNA damage caused by IR is an important activator of microglia. High LET directly ionizes DNA molecules, while low LET tends to indirectly damage DNA through ROS and free radicals originating from water radiolysis [6,66,67]. Damaged DNA can quickly trigger the activation of transcription factors such as nuclear factor κB (NF-κB), cAMP response element-binding protein (CREB), and activating protein 1 (AP-1), which control intracellular ROS generation and gene expression of inflammatory factors including IL-1β, TNF-α, cyclooxygenase 2 (COX-2), and monocyte chemoattractant protein-1 (MCP-1/CCL2) [18,68]. While healthy neurons release factors that inhibit microglial activation, radiation-induced damaged or dead neurons reduce this inhibition and increase the production of various chemokines, cytokines, reactive oxygen species, and ATP [13,63,69]. By virtue of abundant receptors on their cell membrane, microglia sense and respond to the changes of “danger” signals in the surrounding environment. For instance, high mobility group box 1 (HMGB1) from neurons or ECs and microglial toll-like receptor 4 (TLR4) expression were upregulated after IR, and the combination of both promoted microglial activation [70,71]. Peripheral immune cells infiltrate the brain tissue following radiation-induced damage to the BBB, and they produce ROS, which in turn activates microglia [3].

Once activated, microglia move towards the injury site, phagocytose apoptotic neurons and cell debris, and produce large amounts of pro-inflammatory mediators [72]. It was shown that following in vivo or in vitro irradiation higher than 7 Gy, microglia produced high levels of ROS, NO, IL-1, TNF-α, IL-6, COX-2, MCP-1, and intercellular adhesion molecule 1 (ICAM-1) [20,62,73,74,75,76]. These pro-inflammatory mediators exacerbated RIBI. Studies in rodents have also shown that activated microglia and TNF-α remained at high levels for at least 6 months after a single high dose of irradiation [77,78]. Such persistently activated microglia continuously release pro-inflammatory factors, which maintain the inflammatory status of the brain microenvironment and further inflict neuronal and progenitor cell death, leading to a vicious circle characterized by microglial activation, release of inflammatory factors, and neuronal death [79]. Persistent inflammation also inhibits neurogenesis in the juvenile and adult hippocampus as X-ray irradiation with 2 Gy at postnatal day 10 induces impairment of neurogenesis, even when animals are six month old [80]. X-ray irradiation of adult mice with 10 Gy suppressed the proliferative capacity of neural progenitor cells (NPCs) in the DG region and induced NPCs to differentiate towards glial cells, which was attributed to an inflammatory response, as aggressive anti-inflammatory strategies partially restored the proliferative capacity of NPC to neurons. Importantly, cognitive and behavioral modifications correspond with increased microglial activation, and administration of anti-inflammatory agents also reduced cognitive impairment in rodent [65,79,80]. It should be noted that factors affecting microglia activation, such as age, gender, environment, and cell interactions with microglial cells, and expression and activation of different receptor on microglia may affect the efficacy of anti-inflammatory strategies [19]. Furthermore, recent research suggests that radiation-induced enhancement of microglial phagocytosis causes alteration of synaptic plasticity, partly resulting in cognitive impairment. These are further discussed in detail in Section 4 and Section 5.

### 3.2. ROS/RNS Production and Oxidative Stress

A delicate balance between reactive oxygen/nitrogen and antioxidants is essential for the maintenance of normal physiological function of the CNS. In RIBI, the disruption of equilibrium often means the excessive accumulation of ROS/RNS in the cell, leading to lipid peroxidation, protein degradation, and DNA damage reactions [81]. Microglia respond to the pathogen- and stressors-associated molecular patterns through the production of ROS and protect normal tissues from insults [81,82]. However, high doses of IR induce excess ROS production, which is further amplified with increasing radiation doses [83,84].

Enzymatic and non-enzymatic reactions are the main pathways of ROS production. After radiation exposure, non-enzymatic ROS are generated in large amounts along with mitochondrial respiration of microglia [84]. NADPH oxidase (NOX) consists of Nox1 to 5 and dual oxidases 1 and 2 and promotes enzymatic ROS production in most cells [85]. Microglia express high levels of NOX, particularly NOX-2 [86]. NOX-2 expression is significantly elevated in the brain within hours after IR, and the NOX-2 inhibitors apocynin and diphenylene iodonium, or the neutralizing antibody to NOX-2 significantly reduced radiation-induced ROS production [87]. In irradiated microglia, NOX activation-mediated ROS production is modulated by the mitogen-activated protein kinases (MAPKs) signaling cascade through phosphorylation of c-Jun, a component of AP-1 transcription factors [88,89]. Mitochondrial translocator protein 18 kDa (TSPO) is located on the outer mitochondrial membrane, similar to NOX2, and it is upregulated in reactive microglia [90,91]. It has been shown that gamma irradiation with 2 Gy upregulated TSPO expression in primary microglia [90]. TSPO is associated with ROS generation and subsequent oxidative stress. Stimulation of primary microglia with TSPO typical ligands PK11195 and Ro5-4864 induced ROS production, and prior application of a NOX inhibitor reversed this effect [92]. Two recent studies have revealed more details on this interaction between TSPO and NOX in microglial ROS production [93,94]. In mice with selective deletion of TSPO in microglia, it has been demonstrated that TSPO-mediated ROS generation is Nox1 dependent in reactive microglia, and an increase in cytosolic calcium concentrations is necessary for functional coupling between TSPO and NOX-1 [93]. On the other hand, TSPO interacts with NOX2 subunits gp91Phox and p22Phox in resting microglia. This interaction is disrupted after endotoxin exposure, resulting in upregulation of TSPO at the mitochondria and plasma membrane, which provided a biophysical foundation for their interaction that regulates ROS production under radiation conditions [94]. In addition to affecting mitochondria-associated oxidative stress, TSPO also affects the microglial genomic function. TSPO is involved in inflammatory transcriptional programs, including NLRP3 inflammasome, NF-κB, and MAPK [95,96,97], leading to the release of multiple cytokines. In primary human, mouse, and rat microglia, PK11195 has been shown to inhibit LPS-induced production of inflammatory factors such as TNF-α, IL-6, and NO [98,99,100,101]. Recently, treatment of microglia with the new generation TSPO ligands 2-cl-mgv-1 and mgv-1 also reduced the production of COX2, iNOS, and NO after LPS stimulation [101,102].

Another cause of ROS accumulation is dysregulation of complex antioxidant systems. Microglia contain superoxide dismutase, catalase, and NADPH-regenerating enzymes, as well as a high concentration of glutathione and enzymes necessary to generate glutathione, which confer high antioxidant activity to these cells. A single dose of more than 2 Gy significantly reduces the activity of antioxidant enzymes, including superoxide dismutase (SOD), glutathione, and catalase [103,104,105].

ROS derived from NOX and mitochondria may underlie radiation-induced excessive inflammation. There is ample evidence that enzymatic ROS promotes the release of proinflammatory factors in microglia, and both direct inhibition of NOX-2 and elimination of NOX-2-dependent ROS production reduce the expression of pro-inflammatory factors in microglia [106,107]. Indeed, as the first messenger of intercellular communication, ROS released by microglia change the redox state of adjacent cells [108,109]. On the other hand, increased ROS, as second messengers, through affecting the activation of kinase pathways and transcription factors, promote microglial immune activation, and subsequently amplify and perpetuate neuroinflammation [110]. For instance, ROS could directly react with IκB kinase, which inhibits NF-κB activity or catalyzes the release of NF-κB subunit from the IκB binding state through redox activation of upstream kinases, thereby initiating the expression of pro-inflammatory genes in microglia [111,112]. Co-incubation of BV-2 cells with PPARδ agonist suppressed the radiation-induced increase in intracellular ROS generation to reduce NF-κB and AP-1 activation and inflammatory factor gene expression [18]. Limiting mitochondrial ROS accumulation with mitoTEMPO suppressed MAPKs activation and nuclear translocation of NF-κB, accompanied by the reduced expression of different proinflammatory factors, such as TNF-α, IL-1β, IL-6, iNOS, and COX-2 [113]. The peak of radiation-induced ROS production precedes IL-1, TNF-α, COX-2, and MCP-1 in microglia [18,20]. After fractionated whole-brain irradiation, the production of ROS peaks at 4 h after radiation, whereas protein levels of TNF-α and MCP-1 are significantly increased at 8 h after radiation [87]. Therefore, radiation-induced ROS may be an important cause for the subsequent occurrence of pro-inflammatory events in RIBI.

### 3.3. Regulation of BBB Integrity

BBB is composed of ECs, basal lamina, and astroglial end-feet. With highly selective permeability, BBB selects and controls the entry of most molecules from the circulating blood into the CNS [6]. In physiological conditions, perivascular microglia physically contact with ECs and monitor the passage of blood solutes through the BBB. Microglia also express tight junction protein claudin-5 to maintain tight-junction integrity between ECs [114,115]. So far, only a few studies have investigated the interaction of microglia with the BBB after IR exposure.

Following a single whole-brain irradiation with 20–60 Gy, an acute increase in BBB permeability was detected with the application of BBB permeable tracers. This BBB collapse is reversible and can be restored within weeks [64,116]. Although radiation at a dose of 10 Gy does not significantly damage BBB in mice, minor BBB permeability alterations may occur [117,118]. Fractioned-irradiation with a total dose of 40 Gy (2 Gy per fraction) for four weeks results in an increase in BBB permeability, which may last for 180 days [119]. Moreover, as a major trigger of increased BBB permeability, radiation-induced EC apoptosis increases with time and dose [3]. Irradiated ECs secrete cellular signals through the NF-κB pathway to activate microglia and attract microglia migration toward adjacent blood vessels [120,121]. Irradiation of isolated and co-culture systems show that astrocyte activation requires microglia-derived factors, including prostaglandin E2 (PGE2) [62]. An in vivo experiment also confirmed that radiation-induced astrocyte activation is medicated by C1q, which is produced by microglia [71]. In such a way, microglia and astrocytes exert synergistic effects to co-release proinflammatory cytokines, such as TNF-α and IL-6, which stimulate surviving ECs to upregulate their intercellular adhesion molecule 1 on the luminal surface of blood vessels [7,64,122]. Irradiated microglia can produce ICAM-1 directly or release TNF-α and IL-6 to activate astrocytes to produce ICAM-1 [123]. In response, peripheral leukocytes are recruited onto ECs and, along with microglia, secrete matrix metalloproteinases (MMPs) that break down the BBB, which then allows peripheral immune cells to enter the brain parenchyma and exacerbate brain damage [7,124]. In addition, activated microglia could downregulate claudin-5 expression via TNF-α production, which contributed to the radiation-induced early BBB disruption [64]. Anti-TNF-α treatment reduced BBB permeability and ICAM-1-dependent leukocyte adhesion in mice exposed to X-ray radiation with 20 Gy [125].

### 3.4. Immune Cell Infiltration in the Brain

Although microglia are innate immune cells in the brain, peripheral immune cells migrate into the brain due to disruption of BBB after high doses of ionizing radiation [126]. CD3+ cells infiltrate to brain tissues within 7 days after irradiation and stay there for 12 months, whereas penetration by CD11c+ and MHC II+ cells occurs at the late stage after 7 days. However, distinguishing peripherally infiltrated immune cells from resident microglia is difficult, as these two groups of cells express many identical immune markers, such as CD11c, CD 68, and MHC II [127]. With the identification of characteristic markers for microglia, the application of transgenic and bone marrow chimeric animals and experimental techniques, such as flow cytometry and two-photon imaging, identification, and functional investigation of infiltrating immune cells became feasible. Using bone marrow chimeric mice, the dose-dependent recruitment of bone marrow-derived (BMD) cells and their differentiation into inflammatory cells and microglia were demonstrated in the irradiated brain region [128]. This recruitment can persist up to 6 months after irradiation with doses above 15 Gy [129]. Mildner et al. identified a specific monocyte population that penetrated the brain and presented a microglia phenotype after cranial radiation [117]. Even in the absence of radiation-induced detectable BBB damage, blood-derived macrophages are recruited to the brain and express CX3CR1, a marker unique to microglia [117,118]. This recruitment without BBB damage may be a consequence of increased levels of adhesion molecules, chemokines, and their receptors associated with immune cell infiltration in the postradiation brain [118,130]. Among them, CCL2-CCR2 signaling has been shown to participate in this process. Irradiated microglia can secrete CCL2, but barely express CCR2 [131,132]. Several studies have reported that high doses of IR (≥9 Gy) caused increased levels of CCR2 + macrophages and CCL2 in the mouse brain parenchyma [71,118,133]. CCR2 deficiency reduced colonization of BMD immune cells into the brain 6 months after cranial radiation [130]. Interestingly, under relatively low-dose irradiation (doses below 2 Gy), CCR2 knockout mice exhibited preservation of survival NPCs in the hippocampus and improvement of spatial memory and learning deficits [134]. After a high dose of radiation (10 Gy), this protection afforded by CCR2 deficiency against cellular and behavioral deterioration was also identified [130]. These studies suggest that infiltrating cells may potentially exacerbate RIBI, although work remains to distinguish resident microglia and infiltrating immune cells. The expression of CCR2 in NPCs, granule cells, and pyramidal neurons after irradiation may also shift the researcher’s attention to the role of infiltrating cells in RIBI [135]. Dietrich et al. demonstrated that BMD macrophages and monocytes were chronically increased in the irradiated site and communicated with the cellular microenvironment where they existed perpetually, which reduced the inhibition of radiation on neuro-glial progenitor cell proliferation and improved cognitive function [136]. In summary, there are only a few RIBI models that investigate the functional roles of infiltrating cells. Since treatment that pharmacologically targets CNS microglia to prevent RIBI may also affect the survival, proliferation, and functional transitions of peripheral immune cells, further examination of the contributions of infiltrating cells to RIBI and their recruitment mechanisms is highly warranted.

## 4. Modulation of Microglia for RIBI Therapy

**Figure 2:**
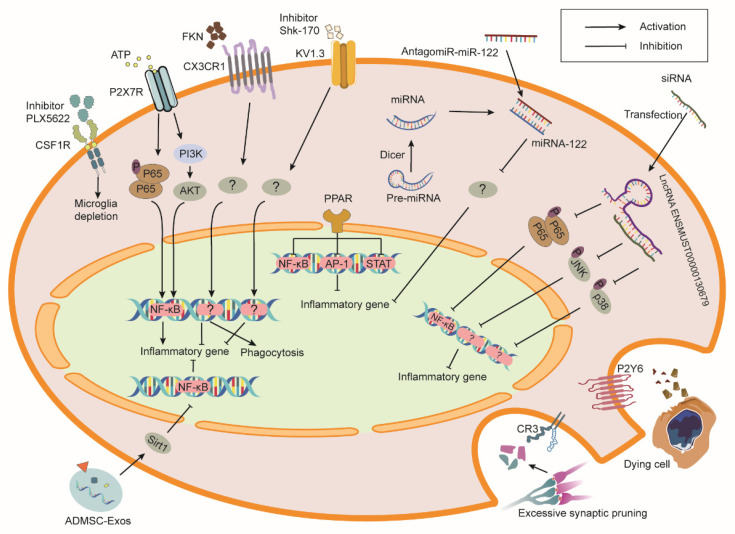
Schematic overview of receptors and exogenous molecules that modulate microglia phenotypes and functions in RIBI. Colony stimulating factor 1 receptor (CSF1R) blockade causes microglial death. Inhibition of P2X7 receptor (P2X7R) and KV1.3 channel or activation of (C-X3-C motif chemokine receptor 1) CX3CR1 and peroxisome proliferator-activated receptor (PPAR) prevent inflammatory gene expression in microglia. Activation of P2Y6 receptor (P2Y6R) and C3 receptor (CR3) mediate the phagocytosis of dying cells and synaptic components by microglia, respectively. Moreover, the introduction of exogenous molecules such as microRNA (miRNA), long non-coding RNAs (LncRNA), and extracellular vesicles (EVs) also enhance the therapeutic efficacy of RIBI. ATP, adenosine triphosphate; P, phosphorylation; NF-κB, nuclear factor κB; PI3K, phosphoinositide 3 kinases; AKT, protein kinase B; FKN, fractalkine; Shk-170, Stichodactyla helianthus-170; Pre-miRNA, precursor-miRNAs; siRNA, short interfering RNA; AP-1, activator protein 1; STAT, signal transducer and activator of transcription; Sirt1, sirtuin 1; ADMSC-Exos, exosomes derived from adipose mesenchymal stem cells.

Many different biochemical mediators, their receptors, and downstream signaling pathways are involved in microglial reaction to RIBI (Figure 2). Inhibition or activation of these pathways may prevent RIBI (Table 1).

### 4.1. Colony Stimulating Factor 1 Receptor (CSF1R)

Colony stimulating factor 1 (CSF1) and its receptor CSF1R are key regulatory signals in myeloid cell development [137]. CSF1R is mainly expressed by microglia in the CNS. This signaling axis regulates the proliferation, differentiation, and survival of microglia, which is crucial for early brain development. Mice with mutations in the CSF1R gene exhibit loss of microglia, severe brain defects, and a shortened life span [138,139]. Genetic ablation or pharmacological approaches to inhibit CSF1R related signal transduction pathway leads to the almost complete elimination of microglia [140,141].

Depletion of microglia with a dietary inhibitor of CSF1R reduced the release of pro-inflammatory factors and the number of activated microglia in the hippocampus of mice after brain injury induced by acute ionizing radiation [141]. Animal memory deficits, spatial exploration, and fear extinction deficits were also improved one month after the treatment. Similarly, in the mouse model of fractionated whole-brain cesium-137, helium, or cosmic irradiation, CSF1R blockage attenuated neuroinflammation and improved learning and memory [142,143,144]. Neuroanatomical study indicated that IR could affect the density and integrity of synapses. For instance, exposure to doses higher than 1 Gy of γ- or X-rays causes a prolonged decrease in density, length, and area of dendritic branches, as well as the number of dendritic branch points in the hippocampus [145,146,147], whereas developing hippocampal dendritic spines and excitatory synapses increased one hour after radiation [147]. High-LET heavy particle irradiation or low-LET γ irradiation also induced alterations of synaptic protein, post-synaptic density protein 95 (PSD-95), and synapsin-1 [143,144,146,148,149], which were involved in spatial choice and recognition memory, respectively [150,151]. However, microglial depletion prevented loss of dendritic spines, especially matured mushroom spines, and improved learning and memory [142]. The rescue of spine density may result from diminished phagocytic function of repopulated microglia, as microglia mediate synapse elimination by the complement system, and repopulated microglia reduce the phagocytic marker complement component 5a receptor 1 (C5aR) and lysosomal associated membrane protein 1 (LAMP-1) [144]. These findings strongly support microglial involvement in radiation-induced synaptic damage and cognitive impairment or RIBI.

As mentioned earlier, microglia play an irreplaceable role in maintaining physiological functions of the nervous system, such as supporting neuronal generation, clearing cell debris, promoting myelination, pruning synapses, and refining neural circuits. Interestingly long-term depletion of microglia by CSF1R inhibitors does not affect the cognitive function or basic motor ability in mice [141,152]. Morphological and electrophysiological features of neurons also remain relatively normal [143]. Even prolonged depletion of microglia limited the expression of proinflammatory chemokines, cytokines, and reactive oxygen species (ROS), thereby attenuating chronic neuroinflammation in mice [153]. Furthermore, microglia proliferate rapidly and refill into the brain after discontinuation of the inhibitors, and the re-proliferated microglia present fewer pro-inflammatory phenotypes [143,152,154,155]. Nevertheless, elimination of microglia early in stroke enlarged the size of the infarct [156]. The depletion of microglia before spinal cord injury caused the mice to show more severe motor impairment [153]. In mouse models of Parkinson’s [157] and viral encephalitis [158], microglia depletion was also observed to have a counterproductive effect. Therefore, more studies are required to elucidate the time windows, mechanisms, and safety of CSF1R inhibition therapy. Notably, neural progenitor cells and cortical neurons also express CSF1R. Whether the non-binding effect caused by the depletion of microglia cells with CSF1R inhibitors will affect the function of these cells remains to be further investigated.

### 4.2. Complement Receptors and Complement Components

The complement system is an innate immune-surveillance system and consists of more than 40 serum proteins, cell surface receptors, and regulators [159]. It also performs non-immune functions in the CNS, for example, marking synapses for elimination [160]. Microglia can express complement components C1q and complement receptor-1 and, upon activation, facilitate the presentation of their own complement receptor-3 (CR3) and upregulate C3 release [161,162,163]. As such, activated microglia are bound to enhance complement cascade and vigorous production of immune effector molecules, leading to immune reactions, inflammatory processes, and engulfment of substances flagged by complement [48,160].

Early after exposure to IR, the complement proteins C1q, C3, C3a, and C5aR increase dramatically in the hippocampus of mice [145,163,164,165,166]. C1q is an upstream component of the complement cascade, and its biosynthesis in the brain is mainly dependent on microglia [163]. In order to avoid the effects of global knockout or pharmacological inhibition on the peripheral complement cascade in vivo, Mineh and colleagues evaluated the contribution of microglial-specific proximal cascade component C1q on RIBI by selective knockout of C1q in mouse microglia [71]. Four weeks following exposure, C1q-deficient mice showed lower levels of inflammatory factors and activation of microglia and astrocytes than did wild-type (WT) mice after radiation. C1q deficiency also prevented C3 accumulation on astrocytes and downregulated the protein levels of C5aR1 and microglial TLR4, partially accounting for the attenuated inflammatory response in the hippocampus of C1q-deficient mice, as C5aR1 signaling promotes microglial inflammatory polarization and synergizes with the TLR4 in the acute inflammatory response to endotoxin [167,168]. These changes were followed by a reduction in dendric spine loss and high immunoreactivity of synaptophysin and synaptic vesicle glycoprotein 2, suggesting the preservation of the synaptic structure, which coincided with better performance in the place recognition test for mice lacking C1q compared to WT mice [71].

C3, a downstream component of the complement cascade, integrates three pathways of the complement system (classical, alternative, and lectin pathways). Several activated fragments (C3b, iC3b, C3c) of C3 cleaved by proteolysis acutely promote the extent and duration of the inflammatory response and remarkably enhance phagocytosis of microglia expressing multiple complement receptors in the context of neurological disorders [48,169]. Hinkle et al. showed that the genetic deletion of C3 rescues proliferating cell loss in the hippocampus of mice exposed to a single γ-irradiation of 8 Gy at postnatal day 10, as well as cognitive performance in hippocampal-dependent behavioral tasks [165]. Moreover, pharmacological blockade of the C3 receptor (CR3) improves cognitive dysfunction and reduces spine loss and the number of microglia CD11 in male mice exposed to 10 Gy γ irradiation [166]. This reduction also occurs in irradiated mice with CR3 knockout [145]. Strikingly, two studies reported that only male but not female mice exhibited reduced dendritic spine density and behavioral defects [145,166]. Consistent with these results, mice that received mixed or single-particle-radiation also exhibited sex-dependent synaptic alterations and cognitive impairments [170,171]. Because higher phagocytosis activity of microglia appears in the male mouse brain than in females after radiation [144,170], these sex-specific findings again underline the possible relationship between microglia phagocytosis, impaired synaptic structure, and cognitive deficits.

Previous studies have shown that C1q and C3 were located at synapses, directing microglial pruning of synapses during the development of neural circuits [48,160]. In a mouse model of multiple sclerosis (MS), C1q and activated C3 appeared in microglial processes and lysosomes, suggesting that synaptic components were phagocytosed by microglia [172]. Additionally, synapse loss and cognitive dysfunction are restored in AD model mice when C1q, C3, or CR3 are inhibited [173,174]. Taken together, these results suggest that radiation-induced C1q production by microglia may activate the complement cascade and multiple complement proteins and receptors, including C3 and CR3, which is a deviation from their normal expression pattern, thus mediating microglial engulfment of synapses and inflammatory response in the hippocampal microenvironment, ultimately leading to hippocampal-dependent cognitive impairment [145,163,165,166].

### 4.3. Purinergic Receptors

In RIBI, ATP is released through lytic, i.e., cell death, and non-lytic, such as exocytosis, ion channels, transporters, and lysosome pathways [175]. Large amounts of ATP bind to purinergic receptors on the surface of neurons and glial cells, especially microglia, as a “danger” signal, prompting microglia to release pro-inflammatory mediators, leading to neurotoxicity [69,176]. A positive correlation was found between elevated ATP levels and the severity of RIBI and the levels of inflammatory factors (COX-2, IL-6, and TNF-a) in CSF [69]. Microglia sense the changes of ATP and its derivatives in the surrounding environment by P2X and P2Y purinergic receptors [175]. Two studies have investigated the role of the microglial P2X7 receptor (P2X7R), an ATP-gated cationic channel and the P2Y6 receptor (P2Y6R), a metabolic G protein-coupled receptor in RIBI [177].

β-ray radiation with 10 Gy is sufficient to induce increased P2X7R expression both in cultured and in vivo microglia [69]. Inhibition of P2X7R with a P2X7R antagonist or short interfering RNA (siRNA) significantly reduced the number of activated microglia, the secretion of inflammatory factors, and the loss of neurons, indicating that the ATP-P2X7R signaling axis is involved in radiation-induced microglial activation and brain injury [69]. The mechanism involves NF-κB and PI3K-Akt signaling pathways downstream of P2X7R. Furthermore, reducing extracellular ATP levels or antagonizing P2X7R may result in the receptor changing from an inflammation-induced phenotype to a scavenger receptor phenotype, characterized by increased phagocytic activity and decreased lysosomal pH. The acidification of lysosomes is indicative of a high rate of clearance for endocytosed material [178,179,180,181]. Importantly, microglia with enhanced phagocytosis prevent secondary inflammatory responses by clearing debris, neurite outgrowth inhibitor (Nogo)-A, and potentially toxic products [182,183,184]. Therefore, further investigation of the regulatory mechanism of the P2X7R receptor on this clearance may help to establish novel therapeutic targets for neuroinflammation caused by increased waste material in RIBI.

Contrary to P2X7R, blockade or knockout of P2Y6R appears not to affect the release of inflammatory mediators from microglia [182,185]. Radiation has been found to enhance microglial phagocytosis in vitro and in vivo, accompanied by an increase in P2Y6R expression [182]. Xu et al. reported that P2Y6R activation mediates increased phagocytosis of microglia via the Ras-related C3 botulinum toxin substrate 1 (Rac1)-myosin light chain kinase (MLCK) signaling pathway, which allows microglia to clear apoptotic neurons and myelin debris Nogo-A, thereby promoting remyelination and microenvironment recovery in the RIBI animal model [182].

### 4.4. CX3CR1

FKN, the ligand of CX3CR1, is expressed mainly in neurons, with some from microglia, astrocytes, and ECs as well [42,186]. Fractalkine binding to CX3CR1 can maintain microglia in a quiescent state and inhibit the release of inflammatory cytokines. Therefore, in animal models with neurodegenerative diseases and traumatic brain injury, the impaired FKN/CX3CR1 signal axis is often accompanied by abnormal activation of microglia and the deterioration of the disease [187,188,189,190].

Recently, the importance of the FKN/CX3CR1 axis was confirmed in radiation-induced microglial activation and brain injury [20]. Following IR, artificially increased FKN reduced the release of IL-1β and TNF-α in BV-2 cells. In mice exposed to whole-brain radiation, increased FKN promoted microglia to shift from neurodestructive to neuroprotective via FKN lentivirus to prevent the decline of the number of neural stem cells in the hippocampus. In contrast, the knockdown of CX3CR1 resulted in the partial reversal of FKN-mediated neuroprotection. Considering that CX3CR1 is exclusively expressed in microglia in the CNS, showing exactly how FKN/CX3CR1 regulates microglial activation may provide a microglia-specific means to control the inflammation in RIBI.

### 4.5. Peroxisome Proliferator-Activated Receptors (PPARs)

PPARs are a class of ligand activated receptors in the hormone superfamily of nuclear receptors, including three subtypes of PPAR-a, PPAR-β/γ, and PPAR-δ [191]. It is initially believed that PPARs signaling pathway is involved in cell metabolism. They can be activated by some natural ligands, such as certain essential fatty acids, phytanic acid, eicosanoids, etc., thereby regulating fatty acid oxidation, glucose homeostasis, and various other metabolic pathways [192]. Studies on PPAR agonists have shown that PPAR agonists inhibit the activation of inflammatory transcription factors, such as NF-κB, AP-1, and STAT [83,193,194,195] and enhance the expression of catalase and superoxide dismutase [195,196]. Thus, activating PPARs can exert neuroprotective effects in a range of neurological disorders by regulating oxidative stress and inflammatory processes [197,198,199].

Microglia can express three PPAR subtypes including PPAR-a, PPAR-β/γ, and PPAR-δ [200]. Pretreatment of BV-2 cells with PPARα agonist GW7647 or fenofibrate decreased the mRNA level of TNF-α and IL-1 and the protein level of COX-2 induced by radiation, which depended on PPARα negatively regulating the activity of AP-1 and NF-κB by inhibiting nuclear translocation of the p65 subunit and phosphorylation of nuclear c-Jun, respectively [83]. Administration of fenofibrate promoted newborn neuron survival and prevented microglial activation in mouse hippocampus after whole-brain radiation with 10 Gy [201]. Furthermore, radiation-induced perirhinal cortex-dependent cognitive impairment was prevented, although fenofibrate failed to significantly ameliorate these pathological characteristics in the hippocampus of rats receiving fractionated whole-brain radiation [78]. The study on PPARδ showed that pretreatment BV-2 with PPARδ agonist also reduced radiation-induced increases in TNF-α and IL-1 mRNA, COX-2 protein, and ROS production [18]. This inhibition of proinflammatory factors was attributed to the weakened DNA-binding activity of NF-κB via the physical interaction between PPARδ and the P65 subunit, and to the inhibition of PKCα/MEK1/2/ERK1/2/AP-1 pathway activation [18,202]. Likewise, dietary PPARδ agonist GW0742 downregulates the expression of neuroinflammation and the number of activated microglia in the hippocampus of the whole-brain irradiated mouse [203]. PPAR-γ is the highest expression subtype in microglia. Once activated by the agonist pioglitazone, PPAR-γ blocked p38 MAPK signaling and in turn led to decreased microglia activation and lower levels of STAT-1 and NF-κB activation [204,205]. Continuous administration of pioglitazone before, during, and for 4 or 54 weeks after exposure significantly reduced short-term or long-term hippocampus-dependent cognitive impairment [206].

Although in vitro experiments highlighted the role of agonists of PPARs in attenuating the radiation-induced release of proinflammatory factors from microglia [18,83], it remains unclear whether the effects of PPAR agonists result directly/indirectly from microglia in rodent models, as PPARs is widely expressed by a variety of cell types in the CNS [207]. In addition, neither PPARα nor PPARδ activation improved hippocampal-dependent cognitive impairment in rats [78,203], which is not compatible with reduced neuroinflammation and fewer activated microglia in the hippocampus. Therefore, further research is needed to explore the effect of PPARs on other cells in the CNS and clarify whether the effects of PPAR activation are subtype-dependent in the RIBI.

### 4.6. Kv1.3 Channel

Kv1.3, a voltage-gated potassium channel with six transmembrane segments, was first found in human T lymphocytes in 1984 [208]. Since then, it has been realized that Kv1.3 is highly expressed in a variety of immune cells, including microglia cells, and that Kv1.3 plays an important physiological role in regulating microglia membrane potential, calcium signal transduction, cytokine production, and proliferation [209,210]. Many studies have shown that genetic deletion or pharmacological blockade of Kv1.3 attenuates the activation of microglia cells and the release of pro-inflammatory factors following stimulation with Aβ, ischemic stroke, aggregated α-synuclein (αSynAgg), or LPS, suggesting that the expression of voltage-gated potassium channel Kv1.3 is one of the prerequisites for microglia activation [208,211,212,213]. Hence, the increased expression of Kv1.3 has been observed in AD, ischemic stroke, and Parkinson’s disease, and with the pharmacological or genetic inhibition of Kv1.3, both pathological and neurological outcomes have improved in relevant animal models [208,212,213].

In a cell model of radiation exposure, transfection of microglia with kv1.3-specific shRNA restrained the radiation-induced increase of COX-2 and IL-6 mRNA and protein. Blockade of Kv1.3 with Stichodactyla helianthus (Shk)-170, a selective peptide inhibitor of Kv1.3, inhibited the apoptosis of primary hippocampal neurons induced by irradiated BV2 microglia in the co-culture system [74]. Moreover, mice injected intraperitoneally with Shk-170 at 3 days post-irradiation also exhibited significantly reduced inflammatory factors and microglial activation but increased proliferation of neural progenitor cells in the hippocampus [74]. Studies have shown that the neuroprotective effect of the Kv1.3 blocker is achieved partly through disrupting P2X4-mediated calcium influx, thus reducing microglia activation [214,215]. Moreover, microglial Kv1.3 blockade also reduced αSynAgg, LPS, or Aβ-induced p38MAPK phosphorylation and NF-κB activation [212,213]. However, the basic molecular mechanism and the exact signal transduction pathway of limiting RIBI by Kv1.3 blockade remain unclear.

### 4.7. MicroRNAs (miRNAs)

miRNAs are single-stranded noncoding RNAs with 20 to 25 bases. By binding to the 3’-untranslated region of the target messenger RNA, miRNAs promote the direct degradation of mRNA by nuclease or prevent the translation process of mRNA, thereby extensively regulating gene expression at the post-transcriptional level [216,217]. MicroRNA sequencing has increasingly demonstrated a variety of differentially expressed miRNAs involved in the development of RIBI by affecting multiple processes, such as inflammatory responses, DNA damage, apoptosis, proliferation, etc., and the dysregulation of some miRNAs may lead to overactivation and increased radiation sensitivity of microglia [218,219,220,221].

Dicer has endoribonuclease activity and can cleave miRNA precursors into mature miRNAs [222]. Bioinformatics analysis of miRNA suggests the loss of some stable maintenance and anti-inflammatory miRNAs in Dicer-deficient microglia [223]. In line with the prediction, Dicer-deficient microglia exhibit DNA damage and spontaneous activation in perinatal period and excessive activation stimulated by peripheral toxins in adulthood [222,224]. Despite the strong resistance of microglia to IR [76], selective deletion of Dicer in mouse microglia produced significantly increased DNA damage and apoptotic microglia after radiation, implicating a critical role for miRNA in DNA repair and maintenance of the resting state for microglia [223].

miR-741-3p is a member of the fragile-X miRNA cluster [225] and was dramatically upregulated in rat models of nonalcoholic fatty liver disease [226] and attention-deficit/hyperactivity disorder [227]. A previous study showed protection of miR-741-3p against radiation-induced injury of bone mesenchymal stem cells [228]. Recently, Ou et al. indicated that miR-741-3p expression is significantly upregulated in the mouse hippocampus from 1 day to 6 weeks after 30 Gy radiation. Furthermore, nasal delivery of antagomiR-741 (an inhibitor of miR-741-3p) improved the pathological characteristics of RIBI, presenting less neuronal injury and microglia-related inflammation compared with the radiation-only group, which may be responsible for the improvement of spatial memory at 6 weeks after radiation [229].

RNA sequencing both in blood samples of nasopharyngeal carcinoma patients undergoing radiotherapy and in hippocampus of mice with RIBI revealed significant upregulation of mir-122-5p [230]. Inhibition of miR-122-5p expression in the brain through nasal delivery of antagomiR-122-5p resisted hippocampal neuronal damage and cognitive impairment, and these processes may be achieved partly by regulating inflammatory factors generation and microglial activation [230]. AntagomiR-122-5p also decreased radiation-induced release of TNF-α, IL-6, and IL-1ß in BV-2 cells in vitro and thereby repressed the apoptosis of co-cultured SH-SY5Y cells. In addition, miR-122-5p can negatively regulate relative mRNA and protein expressions of tensin 1 (TNS1) in irradiated human microglia clone 3 (HMC3), and whether TNS1 is a downstream target of miR-122-5p to regulate microglial polarization in RIBI requires further validation [230].

In a mouse receiving 10 Gy of γ irradiation, miR-124 overexpression through hippocampal injection of adenoviral particles with miR-124 sequences reduced CD68-positive microglia and ameliorated cognitive impairment after five weeks of radiation [231]. In other models of CNS diseases, multiple signaling, including TLR4 [232], mammalian target of rapamycin (mTOR) [233], CCAAT enhancer binding protein (C/EBP)-α [234], and vesicle-associated membrane protein (VAMP3) [235], could be modulated by miR-124 to promote M2 polarization of microglia and improve neuroinflammation. Moreover, miR-124 directly inhibited neuronal autophagy and apoptosis in the ischemic attack [236], as well as neurodegeneration in traumatic brain injury [237]. The roles of miR-124 in mitigating cognitive impairment may therefore involve multiple mechanisms, more than the regulation of microglia-mediated neuroinflammation, and further investigations of miR-124-related neuropathological features may provide novel mechanistic insights into the protective effects of miR-124 against RIBI.

### 4.8. Long Non-Coding RNAs (lncRNAs)

lncRNAs are a class of non-coding RNAs with a length of more than 200 nucleotides [238]. Although lncRNAs lack open reading frames and do not participate in protein coding, some regions of lncRNAs fold themselves or fold the multiple regions together to form secondary and tertiary structures. Such structural diversity contributes to extensive interactions between lncRNA and proteins, DNA, or RNA, which consequently change cellular and molecular functions and affect the disease process [239,240]. Xu et al. showed increased levels of LncRNA ENSMUST00000190863 and ENSMUST00000130679 in BV-2 cells, as well as in primary microglial cells isolated from the hippocampus of mice at 24 h after 10 Gy X-ray radiation [66]. Through siRNA transfection, they demonstrated that increased ENSMUST00000190863 or ENSMUST00000130679 in microglia promoted DNA damage (DDR) and phosphorylation of p65, JNK, and p38, as well as subsequent downstream pro-inflammatory cytokine release after radiation, which resulted in apoptosis of co-cultured neural stem cells [66]. Moreover, radiation increased lipid droplet accumulation within microglia, and almost one-third of the metabolism-associated genes of ENSMUST00000190863 and ENSMUST00000130679 downstream are related to lipid metabolism, raising the possibility of adipogenesis regulation by them. In turn, the microglial inflammatory state, DNA damage response, and intracellular accumulation of lipid droplets all upregulate expression levels of the above-two lncRNAs [66]. This positive feedback relationship may serve to amplify the lncRNA-regulation efficacy in RIBI therapy.

### 4.9. Extracellular Vesicles (EVs)

EVs are lipid bilayer membrane structures secreted by cells in both physiological and pathological conditions. EVs are divided into two broad categories, namely, exosomes and microvesicles (MV), which mediate intercellular communication by carrying genetic material, protein, and lipids to nearby cells and cells far away [241,242]. In radiation-induced cases of cognitive impairment in rodents, hippocampal transplantation of human neural stem cell (hNSC)-derived MV robustly attenuated microglial activation, preserved complexity of neuronal architecture, and improved performance in behavioral tasks at short (6 weeks) or long (6 months) post-radiation times [231,243,244]. Strikingly, besides for the hippocampus, the neocortex and amygdala similarly exhibited a significant reduction in activated microglia via bilateral hippocampal transplantation of hNSC-derived MV [231]. Furthermore, even after unilateral hippocampal transplantation of MV, a trend toward decreased activated microglia and increased spine density was found in the contralateral hippocampus [243], suggesting both local and remote effects of EV therapy. Liu et al. showed that tail-vein injection of exosomes derived from adipose mesenchymal stem cells (ADMSC-Exos) ameliorates the pathological conditions of cranial irradiation, as indicated by reduced neuroinflammation, oxidative stress, and microglial infiltration in the hippocampus. Further analysis also suggested that ADMSC-Exos activated the SIRT-1/NF-κB signaling pathway to inhibit the release of inflammatory factors from irradiated primary microglia [73]. Furthermore, fluorescence co-localization revealed the uptake of GFP-labeled MV by neurons and astrocytes in the hippocampus of rats exposed to X-ray irradiation with 10 Gy, suggesting that EV-mediated signals may also affect other cells types in the brain, not just microglia [243].

Leavitt et al. analyzed and validated miRNAs as major effector components in hNSC-derived EVs against RIBI [231]. In fact, EVs contain a variety of bioactive content, including non-coding RNAs, neurotrophic factors, cell adhesion molecules, and other factors capable of modulating neural activity [242]. Although the precise mechanisms of EV treatment are still unclear, and the current data in RIBI only revealed the combined effect of different factors in EVs, in other models of CNS disorder and injury, preclinical studies have shown the ability of individual factors within EV to suppress the propagation of neuroinflammation, enhance neurogenesis, maintain myelination, and promote the secretion of neurotrophic factors [231,245]. Therapeutic efficacy of EVs may be optimized by targeting specific EV component equipped with neuroprotective miRNA and other effector molecules for the various molecular biological pathways inherent to RIBI pathology.

**Table 1:** Radioprotective effect of targeting different molecules in microglia in radiation-induced brain injury models.

Targets	Animal/Cell Model	Source Dose and Dose Rate	Irradiated Site	Time Point after Radiation	Intervention Effect in Irradiation Models	Reference
CSFR1	C57BL/6J mouse	X-ray with 9 cGy (1.10 Gy/min)	whole brain	3 days, 2 weeks, 6 weeks	CSFR1 inhibition reduces the increase in mRNA of inflammation markers (TLR9, SYK, CCL6, CD14, CLECL5a, TSLP, CCL5) and the number of activated microglia in hippocampus and ameliorates cognitive dysfunction.	[141]
		^4^He particles with 30 cGy (15–25 cGy/min)		4–6 weeks	CSFR1 inhibition ameliorates cognitive dysfunction, reduces activated microglia population, and attenuates the increase in PSD-95 puncta but does not affect morphologic and electrophysiologic features of neurons.	[143]
		^4^He particles with 15 cGy (16.37 cGy/min) 50 cGy (16.95 cGy/min) 100 cGy (18.07 cGy/min)		18–21 days and 90–100 days	CSFR1 inhibition improves long-term cognitive impairment and inflammatory response, decreases C5aR and LAMP-1, and increases synapsin-1.	[144]
		γ ray with three fractions of 3.3 Gy		1, 3 months	CSFR1 blockade reduces the numbers of activated microglia, suppresses monocyte accumulation in brain, and ameliorates cognitive dysfunction.	[142]
C1q	C57BL/6 mouse	γ-ray with 9 Gy (1.2 Gy/min)	whole brain	2, 24, 48 h; 1, 2, 3, 4 weeks	Deletion of C1q in microglia protects synaptic loss and reduces activation of microglia and astrocytes, as well as protein levels of TNF-a, IL-1ß, IL-6, IL-1α, CCL2, IL-18, and TLR4.	[71]
C3	C57BL/6 mouse	X-ray with 8 Gy (2.3 Gy/min)	whole brain	6 h; 7 days; 2, 3, 4 weeks	C3 knockout improves task performance and increases activated microglia and proliferating cells in the granule cell layer.	[165]
C3R	C57BL/6J mouse	γ-ray with 10 Gy (1.17 Gy/min)	whole brain	30 days	CR3 blockade ameliorates behavior deficits in novel object recognition and the Lashley III maze, prevents dendritic spine loss, and increases CD11-positive microglia in hippocampus.	[166]
				30, 45 days	CR3 knockout prevents dendritic spine loss and increases activated microglia in hippocampus.	[145]
P2Y6	Balb/c mouse	β-ray with 30 Gy (3 Gy/min)	whole brain	1, 14, 30 days	P2Y6 receptor antagonism suppresses phagocytosis of irradiated microglia and increases the number of apoptotic neurons.	[182]
	Primary microglia	β-ray with 8 Gy		4, 12, 48 h	P2Y6 receptor antagonism suppresses phagocytosis of irradiated microglia and has no effect on the production of inflammatory mediators (TNF-α, IL-1β, IL-6, iNOS).	[182]
P2X7	Balb/c mouse	β-ray with 30 Gy (3 Gy/min)	whole brain	3, 7, 14 days; 8 weeks	P2X7R blockade reduces the activated microglia population and neuron loss in the cortex.	[69]
	Primary microglia	β-ray with 10 Gy (6 MeV/min)		24, 48 h	P2X7R blockade reduces the activated microglia population and mRNA expression levels of IL-6, TNF-α, and COX-2.	[69]
CX3CR1	C57BL/6J mouse	γ-ray with 10 Gy (2 Gy/min)	whole brain	3, 6, 12, 24, 48, 72 h; 1, 2, 4 weeks	FKN overexpression promotes M2 phenotypic polarization, reverses the reduced neural stem cell in hippocampus, decreases the TNF-α level, and increases the IL-10 level in the blood.	[20]
	BV-2	γ-ray with 10 Gy (2.0 Gy/min)		1.5, 6 h	FKN promotes microglial phagocytosis and M2 polarization, decreases TNF-α and IL-1β mRNA levels, and increases IL-10 mRNA levels. CX3CR1 knockdown reverses these effects.	[20]
PPARα	BV-2	γ-ray with 10 Gy (4.0 Gy/min)		1, 3, 7, 12, 24 h	PPARα activation prevents the increase in IL-1, and TNF-α mRNA levels, and COX-2 protein via inhibition of p65 translocation and jun phosphorylation.	[83]
	129S1/SvImJ mouse	γ-ray with 10 Gy (3.33 Gy/min)	whole brain	1 week, 2 months	PPARα activation promotes newborn neuron survival and prevents microglial activation. PPARα knockout abolishes the neuroprotection of fenofibrate.	[201]
	Fischer 344 × Brown Norway rats	γ-ray with four fractions of 10 Gy (4 Gy/min)	whole brain	26, 29 weeks	PPARα activation prevents perirhinal cortex-dependent cognitive impairment without a decrease in microglial activation and an increase in immature neurons.	[78]
PPARδ	BV-2	γ-ray with 10 Gy (3.56 Gy/min)		30 min; 7, 24 h	PPARδ activation downregulates ROS production, IL-1 and TNF-α expression, and COX-2 and MCP-1 proteins by inhibiting NF-κB and PKCα/MEK1/2/ERK1/2/AP pathways.	[18]
	C57BL/6J	γ-ray with 10 Gy (5 Gy/min)	whole brain	3 h; 1, 2 weeks	PPARδ activation prevents the increase in IL-1 gene expression and pERK protein but does not rescue neurogenesis and hippocampal-dependent cognitive impairment.	[203]
PPARγ	Fischer 344 rat	γ-ray with nine fractions of 5 Gy (4.41 Gy/min)	whole brain	50, 54 weeks	PPARγ activation prevents cognitive impairment.	[206]
Kv 1.3	Balb/c mouse	ß-ray with 30 Gy (3 Gy/min)	whole brain	3, 14 days; 8 weeks	Kv 1.3 blockade prevents neuronal loss and increases activated microglial in hippocampus and cerebral cortex and improves spatial learning and cerebral cortex atrophy in mice.	[74]
	BV-2	ß-ray with 10 Gy (3 Gy/min)		4, 12 h; 1, 2 days	Kv 1.3 blockade or knockdown decreases protein and mRNA level of TNF-α, IL-6, and COX-2 in microglia and inhibits apoptosis of co-cultured primary hippocampal neurons.	[74]
miR-124	C57BL/6J mouse	γ-ray with 10 Gy (2.07 Gy/min)	whole brain	5 weeks	miR-124 overexpression prevents microglia activation and ameliorates cognitive impairment.	[231]
miR-741-3p	C57BL/6J mouse	ß-ray with 30 Gy (2.5 Gy/min)	whole brain	1, 6 weeks	miR-741-3p inhibition resists cognitive dysfunction, hippocampal neuronal injury, and microglia activation and decreases the expression level of IL-6 and TNF-a.	[229]
miR-122-5p	C57BL/6J mouse	ß-ray with 30 Gy (3 Gy/min)	whole brain	6 weeks, 48–50 days	miR-122-5p inhibition prevents cognitive impairment, neuronal damage, microglia activation, and production of TNF-a, IL-6, and IL-1ß in hippocampus.	[230]
	BV-2	ß-ray with 10 Gy		8, 24 h	miR-122-5p inhibition alleviates the decrease in cell viability and increase in the release of TNF-a, IL-6, and IL-1ß in BV2; restores BV2 branching morphogenesis and phagocytosis; and reduces co-cultured SH-SY5Y cell apoptosis.	[230]
lncRNA ENSMUST00000130679	BV-2	X-ray with 10 Gy (2 Gy/min)		1, 24 h	lncRNA ENSMUST00000130679 knockdown suppresses DDR; phosphorylation of p65, JNK, and p38; and release of TNF-a, IL-6, and IL-1ß in BV2.	[66]
lncRNA ENSMUST00000190863	BV-2	X-ray with 10 Gy (2 Gy/min)		1, 24 h	lncRNA ENSMUST00000190863 knockdown suppresses DDR, phosphorylation of p65, and release of TNF-a in BV2.	[66]
hNSC-derived MV	athymic nude rats	X-ray with 10 Gy (1 Gy/min)	whole brain	4–7 weeks	MV transplantation into the bilateral hippocampus reduces the number of activated microglia in the hippocampus, neocortex (layer II/III), and amygdala; recovers the complexity of neuronal architecture; and ameliorates cognitive impairment.	[244]
				1 month	MV transplantation into the unilateral hippocampus reduces the number of activated microglia in the ipsilateral hippocampus; bilateral or unilateral transplantation increases GDNF and restores PSD-95 protein level in bilateral hippocampus; neither bilateral nor unilateral transplantation protects dendritic spine density.	[243]
hNSC-derived EV	C57BL/6J mouse	γ-ray with 10 Gy (2.07 Gy/minute)	whole brain	5 weeks, 6 months	EV transplantation into the bilateral hippocampus prevents microglia activation in the hippocampus and ameliorates cognitive impairment.	[231]
ADMSC-Exos	Sprague–Dawley rats	γ-ray with 30 Gy (1.59 Gy/min)	whole brain	24 h; 3, 7 days	Tail vein injection pf ADMSC-Exos decreases the levels of caspase-3, MDA, 8-OHdG, TNF-α, IL-4, and SIRT1 and promotes recovery of SOD, CAT, IL-4, and IL-10 levels and suppresses microglial infiltration.	[73]
	primary microglia	γ-ray with 30 Gy (3 MeV/min)		24 h	Tail vein injection of ADMSC-Exos decreases the levels of caspase-3, MDA, 8-OHdG, TNF-α, IL-4, and SIRT1 and promotes the recovery of SOD, CAT, IL-4, and IL-10 levels and suppresses microglial activation. The above effects of ADMSC-Exos are inhibited by the SIRT-1 inhibitor EX527.	[73]

## 5. Conclusions and Open Questions

Microglia with complex heterogeneity fulfill a comprehensive and vital biological function in different brain regions and throughout the whole life cycle. High dose irradiation activates microglia rapidly, and these cells undergo functional alterations by detecting subtle changes in the surrounding microenvironment through receptors. Although we described the intervention of pathological evolution of RIBI from a microglial perspective, the mechanisms behind the intervention and its relationship to radiation-induced neurological dysfunctions are far from clarified, which may restrict the development of therapeutic approaches with microglia as a target. Fortunately, preventing the release of inflammatory factors from microglia and potentiating their clearance of toxic debris by targeting microglial receptors and ion channels seems a feasible approach to enhance neuroprotection. Moreover, studies based on non-coding RNAs and EVs offer new potential avenues for RIBI treatment, with fewer side effects. However, these preclinical studies were conducted in tumor-free animal models to investigate the physiologic and mechanistic effects of treatment, which may compromise proper assessment of these treatment effects on RIBI in patients with tumors. Therefore, new therapies need to be evaluated for their interaction with tumor cells to circumvent the impact of confounders before clinical application. Furthermore, most therapeutic effects have been evaluated in male rather than female rodents, but available evidence indicates gender differences in irradiated microglia, including their activation, as well as microglia-mediated inflammation, neurogenesis, synaptic modification, and cognitive impairment [144,145,170,246,247]. Future research should determine whether gender-specific characteristics of microglia influence therapeutic effects of RIBI.

Clinical and preclinical data indicate that brains from aged patients or animals are more likely to develop persistent microglial activation and chronic neurotoxicity compared to those from young after radiation exposure, which may accelerate the progression of aging-related neurodegeneration disease [72,131,248,249]. Furthermore, irradiated microglia exhibit similar characteristics as normal aging ones, including increased expression of aging-related markers, such as senescence-associated-β-galactosidase (SA-β-gal) and p16^INK4a^ protein, and upregulated genes related to chronic inflammation, DNA damage response, and mitochondrial dysfunctions [66,250,251]. Selective removal of senescent microglia or inhibition of radiation-induced aging phenotypes of microglia may prevent the RIBI process and radiation-accelerated brain aging.

Many previous studies focused on the radiation-induced microglia secretory effect, while less work was done on their phagocytosis in RIBI. Further study on surface receptors mediating microglial phagocytosis, such as triggering receptor expressed on myeloid cells-2 (TREM2) and toll-like receptors (TLRs) after radiation exposure, may open novel therapeutic targets for RIBI treatment. Notably, while we have discussed the clearance of toxic substances by P2X7R- and P2Y6R-mediated phagocytosis in the context of RIBI treatment, recent studies reported that blocking P2Y6R inhibited microglia phagocytosis and prevented LPS, amyloid-β protein (Aβ), and tauopathy (tau)-induced neuron loss and death, indicating the possibility for detrimental effects of P2Y6R-mediated phagocytosis [165,252]. Moreover, preservation of synaptic integrity in RIBI was also associated with the reduction of microglial phagocytic activity or the absence of the complement component mediating microglial synaptic engulfment [142,145,165]. From a speculative point view, irradiated microglia may engulf not only apoptotic cells, but also stressed functional neurons as well as partially healthy synaptic components, thus exacerbating brain damage and cognitive impairment. This is supported by recent studies in the rodent models of stroke and AD [185,253,254]. Therefore, therapeutic approaches via the targeting of microglial phagocytosis to control RIBI should be applied prudently. A better understanding of the molecular mechanism of radiation-induced excessive phagocytosis may allow us to manipulate microglia to distinguish normal neurons from abnormal living neurons in order to reduce therapeutic side effects.

## Data Availability

Not applicable.
